# Testing the psychometric properties of the Finnish version of the cross-cultural competence instrument of healthcare professionals (CCCHP)

**DOI:** 10.1186/s12913-019-4105-2

**Published:** 2019-05-08

**Authors:** Laura Hietapakka, Marko Elovainio, Karolina Wesolowska, Anna-Mari Aalto, Anu-Marja Kaihlanen, Timo Sinervo, Tarja Heponiemi

**Affiliations:** 1 National Institute for Health and Welfare (THL), P.O. Box 30, 00271 Helsinki, Finland; 2 University of Helsinki, Helsinki, Finland

**Keywords:** Cross-cultural competence, Emotions, Empathy, Healthcare professionals, Foreign-born, Multicultural, Psychometric properties, Survey

## Abstract

**Background:**

To test the validity of the Finnish version of the Bernhard et al.’s Cross-Cultural Competence instrument of Healthcare Professionals (CCCHP).

**Methods:**

The study sample comprised registered nurses (*N* = 810) from the Finnish “Competent workforce for the future” -project (COPE). Exploratory factor analyses and structural equation modelling were applied to test structural validity of the CCCHP. Internal consistency of the sub-scales was evaluated using the Cronbach’s alphas. Criterion validity was explored in terms of received education for multicultural work, perceived difficulty of patients, and job satisfaction variables.

**Results:**

The revised version of the instrument including four (motivation/curiosity, attitudes, skills and emotions/empathy) of the five original dimensions provided satisfactory psychometric properties (internal consistency, a good model fit of the data). Of the four remaining competence sub-scales, motivation/curiosity, attitudes and emotions/empathy were associated with the amount of received education for multicultural work, and all with perceived difficulty of patients, and all but attitudes with job satisfaction.

**Conclusion:**

This revised Finnish version of the CCCHP provides a useful tool for studies focusing on the healthcare personnel’s cross-cultural competence in delivering effective and culturally sensitive healthcare services for patients from different cultures.

**Electronic supplementary material:**

The online version of this article (10.1186/s12913-019-4105-2) contains supplementary material, which is available to authorized users.

## Background

The global migration increases the amount of culturally diverse populations in many European countries [1]. Challenges, that healthcare professionals across Europe face when treating immigrants from different cultures include, among others, language barriers, different cultural beliefs or expectations for health and healthcare services, traumatic experiences, difficulties in developing trust and increased risk for marginalization [2, 3]. To meet these challenges, professionals need cross-cultural competence which is generally understood as “*the ability to work and communicate effectively and appropriately with people from culturally different backgrounds”* [4]. Cross-cultural competence is considered essential in understanding the cultural context of the client and delivering effective and culturally responsive services to diverse clients [5].

Healthcare organizations, in turn, need to provide the structures and resources for their professionals to provide culturally competent care. Organizations should also be able to evaluate whether the needs of diverse clients are met [6]. Researchers widely agree that cross-cultural competence is a multi-dimensional construct and that competence can be developed over time [4, 5]. Improving cross-cultural competence requires system-level interventions which include developing and testing instruments that properly measure cross-cultural competence and its outcomes among healthcare personnel [7].

One of the main problems in creating and evaluating the cumulating evidence on the determinants and outcomes of cross-cultural competence in healthcare has been the lack of feasible survey instruments for measuring cross-cultural competence among healthcare personnel. Such measure should provide appropriate psychometric properties, including good structural validity of the scales measuring the construct, and acceptable predictive validity of relevant outcomes. Furthermore, the construct and predictive validity should be replicable in independent samples, such as nurses and physicians.

Several instruments measuring cross-cultural competence have been developed during the last 20 years. However, many limitations in these instruments have been noted (e.g. [4, 8–10]). Definitions of cross-cultural competence and its dimensions vary in different instruments and are based on several different theoretical models. Only few of these instruments have been sufficiently tested empirically, many are tested on a relatively small pool of respondents, and most of them have been developed in the United States for a specific group of healthcare professionals (e.g. nurses) or contexts (e.g. primary care) [4, 8]. The Cross-Cultural Competence instrument of Healthcare Professionals (CCCHP) [11] was developed to overcome some of the limitations of previous instruments. The original aim was to create an instrument that is applicable to the European context and to all healthcare professionals and experts, and has acceptable psychometric properties. The original study was conducted in Germany and the instrument was tested with medical students and psychologists in specific therapeutic training.

In this study, we further tested the validity of the CCCHP in another context (Finnish primary and secondary healthcare), including both native and foreign-born nurses. Thus, we were able to provide further evidence for the instruments’ applicability to European context. We examined the psychometric properties of CCCHP, including its construct validity in large independent sample and tested multicultural education received, perceived difficulty of patients (patients who complain, blame and criticize), and job satisfaction as the measures of criterion validity.

We expected that participants that had had some training on multicultural issues would report higher cross-cultural competence than those who had not had such training [12, 13]. Similarly, we assumed that employees who score high on the cross-cultural competence scale would be less likely to experience difficulties with patients and would have better job satisfaction due to better coping possibilities and control experiences as can be expected following the job demand control model [14]. There are studies which show that at least some of the dimensions of cross-cultural competence, such as intercultural sensitivity, may improve the professional autonomy of nurses when caring for culturally and linguistically diverse patients [15].

### The context of the study

The study was conducted in Finland, which has long been a culturally and linguistically homogenous society. However, the number of international migrants coming to Finland has been increasing for the last two decades. As a result, Finland is progressively becoming a multi-ethnic country among many other European countries. At the end of 2017, the proportion of foreign-language speakers in the total population (altogether 5.5 million) was 7 %. The biggest foreign-language speaking groups were Russian, Estonian and Arabic speakers [16].

Recent studies have indicated some discrepancies in access to care and perceived quality of care between immigrants and the majority population [17]. For example, it has been found that immigrants attend age-salient preventive check-ups or screenings less often, and have had significantly lower access to doctors at healthcare centers than the Finns [18, 19]. Somali-born immigrant women have been found to perceive the attitudes of healthcare providers as unfriendly and communication as poor [20]. Similarly, healthcare providers themselves have considered communication with Somali women as problematic [21]. Thus, the need to promote cultural competence in healthcare professionals is recognized as a priority in many countries including Finland [10, 21].

## Methods

### Study population

This report is based on the data collected at the end of 2017 as a part of the ongoing “Competent workforce for the future” -project (COPE). The data were based on a random sample (*n* = 2001) of all registered nurses born in or after 1950 and who were licensed to practice (as either a registered nurse, public health nurse or midwife) in Finland. They also needed to have a postal address in Finland. We also included additional data of all the foreign-trained registered nurses in Finland (*n* = 617) who met the above-mentioned criteria. Thereby, altogether a sample of 2618 nurses was obtained.

We managed to obtain email or postal addresses for 1790 native and 474 foreign-born nurses. An email or postal invitation to an electronic survey with two reminders was sent. A postal questionnaire was sent to those who did not respond to the electronic survey. The questionnaire for nurses was divided into the following parts: (1) the physical and psychosocial work environment, (2) cultural and overall competence, and (3) well-being and health. The questionnaire was developed for the COPE-project and the English version of the questionnaire is published elsewhere [22]. Response rate was 44.3%. A total of 810 nurses responded in Finnish and 759 of them provided full data of the studied variables (missing pattern is reported in the Additional file 1). The study has been approved by the Finnish National Institute for Health and Welfare Ethics Board.

### Measurements

***Cross-cultural competence*** was assessed with a scale developed by Bernhard and colleagues [11]. Overall competence consisted of five dimensions: (1) motivation/ curiosity (nine items, e.g. ‘I find it exciting to treat patients with a migration background’ and ‘The interaction with people from other cultural backgrounds helps me reflect upon my own cultural background’); (2) attitudes (four items, e.g. ‘Institutions and the public pay too much attention to the special wishes of migrants’); (3) skills (five items, e.g. ‘With patients who do not understand [Finnish] very well, I take more time to explain the treatment options to them’); (4) emotions/ empathy (five items, e.g. ‘In my professional interaction with patients with a migration background, I often feel unsure, angry and frustrated’); and (5) knowledge/ awareness (four items, e.g. ‘Within the migrant population, there are hardly any differences in terms of health opportunities and disease risks’). The response format was a 5-point Likert scale, ranging from (1) fully disagree to (5) fully agree. The English version of the cross-cultural competence scale provided by Bernhard and colleagues [11] was first translated to Finnish by a professional translator and then this Finnish version was again back-translated to English by a person who has native skills in both English and Finnish to ensure the quality of the translation.

***Multicultural training*** was assessed with a question “Have you received multicultural training?”. The response format was: “1 = no, 2 = yes, as part of my qualification/degree, 3 = yes, after graduating e.g., workplace training or other further training and 4 = yes, I have participated in a project or development work related to multiculturalism”. In the analyses, the alternatives from 2 to 4 were merged into one.

***Perceived difficulty of patients*** was assessed with a question “To what extent have difficult patients who complain, blame and criticize disturbed, worried or stressed you in your job in the past six months”. The response format was a 5-point rating scale, ranging from (1) hardly ever to (5) very often or continuously.

***Job satisfaction*** was assessed with a question “How well does the following statement describe your work: Generally speaking, I am very satisfied with my job”. The response format was a 5-point rating scale, ranging from (1) fully disagree (5) fully agree.

### Covariates

***Covariates*** included gender, age, work tenure and contacts with patients. Work tenure was measured as the duration of current employment relationship (the response format in this was: “1=less than one year, 2=1-2 years, 3=3-5 years, 4=6-10 years and 5=over 10 years”). Contacts with patients from different cultures was assessed with a question “How often on average do you meet patients from different cultures in your work?”. The response format was “1=daily, 2=weekly, 3=monthly, 4=less than monthly, 5=not at all”. In the analyses, it was classified into three categories: 1 = less than monthly or not at all, 2 = monthly, 3 = weekly or daily.

### Statistical analysis

The preliminary structural analyses were conducted by calculating bivariate correlations between study variables. Factorial validity of the original scales was tested first with exploratory factor analyses (oblimin rotation and OLS with minimum residual solution due to non-normal distribution of items (Table 1) [23] and then structural equation modelling (confirmatory factor analyses with maximum likelihood estimator; [24]). First, we tested the factor structure and number of dimensions using exploratory factor analyses using optimal coordinates-measure, parallel analysis, velicer MAP, eigenvalue 1 and loading structure as a criterion for the appropriate number of factors (loading higher than .35). Second, we tested the structure of the Finnish version using structural equation modelling. Goodness-of-fit of the SEM models was evaluated based on: the chi square test (X2), the root mean squared error of approximation (RMSEA), the comparative fit index (CFI), the Tucker–Lewis Index (TLI), and Akaike’s information criterion (AIC). A non-significant chi-square value indicates that the model is a good fit to the data. RMSEA values of less than .05 and .08 suggest a good and a reasonable fit, respectively. For CFI and TLI, values above .90 and .95 represent an acceptable and a good fit, respectively. AIC is a measure used to compare any models that have the same set of variables. In such cases, the model with the smaller value of AIC will be preferred [25]. Testing the final structure was done in three steps. First, a one-factor-model was estimated where all remaining items loaded on the same underlying dimension (null model). In the second step, a model representing the original theoretical model was estimated, and, in the final step (if needed), the revised version that provided acceptable fit to the data was developed and tested. Internal consistency of the sub-scales was evaluated using the Cronbach’s alphas.

**Table 1:** Normality tests (D’Agostini) of the CCCHP- scale items

Items	skew	Z	*p*-value
mot1	− 1.48	− 13.06	< 0.001
mot2	−1.33	− 12.14	< 0.001
mot3	− 0.24	−2.77	0.006
mot4	−0.5	−5.55	< 0.001
mot5	−0.73	−7.68	< 0.001
mot6	−0.54	−5.95	< 0.001
mot7	−1.13	−10.82	< 0.001
mot8	−0.83	−8.56	< 0.001
mot9	−1.07	−10.41	< 0.001
att10	0.19	2.22	0.026
att11	0.62	6.72	< 0.001
att12	0.09	1.04	0.297
att13	0.01	1.17	0.247
skill1	−0.98	−9.75	< 0.001
skill2	−0.62	−6.68	< 0.001
skill3	−0.56	− 6.1	< 0.001
skill4	−0.66	−7.08	< 0.001
skill5	−0.85	−8.76	< 0.001
emot6	−0.7	−7.42	< 0.001
emot7	−0.48	−5.36	< 0.001
emot8	−0.49	−5.44	< 0.001
emot9	−1.27	−11.77	< 0.001
emot10	−0.29	−3.37	< 0.001
know11	−0.73	−7.69	< 0.001
know12	−0.53	−5.82	< 0.001
know13	0.29	3.39	< 0.001
know14	2.28	16.85	< 0.001

The associations between the measures (dimensions) of cross-cultural competence and criteria variables (a received multicultural education as a potential antecedent and perceived difficulty of patients and job satisfaction as potential outcomes) were examined using linear regression analyses. In the first step, we calculated an unadjusted model, in the second step, we adjusted the associations for age, gender, contacts with patients and work tenure. The normality test of the CCCHP items and competence dimension measures was based on the procedure presented by D’Agostino and co-workers [26]. We used STATA 15 and R statistical packages (Psych, ggplot2, dplyr, ggcorrplot, stargazer, visreg, nFactors, jtools, mice, VIM, car, lavaan, qgraph) for the statistical analyses.

## Results

**Fig. 1:**
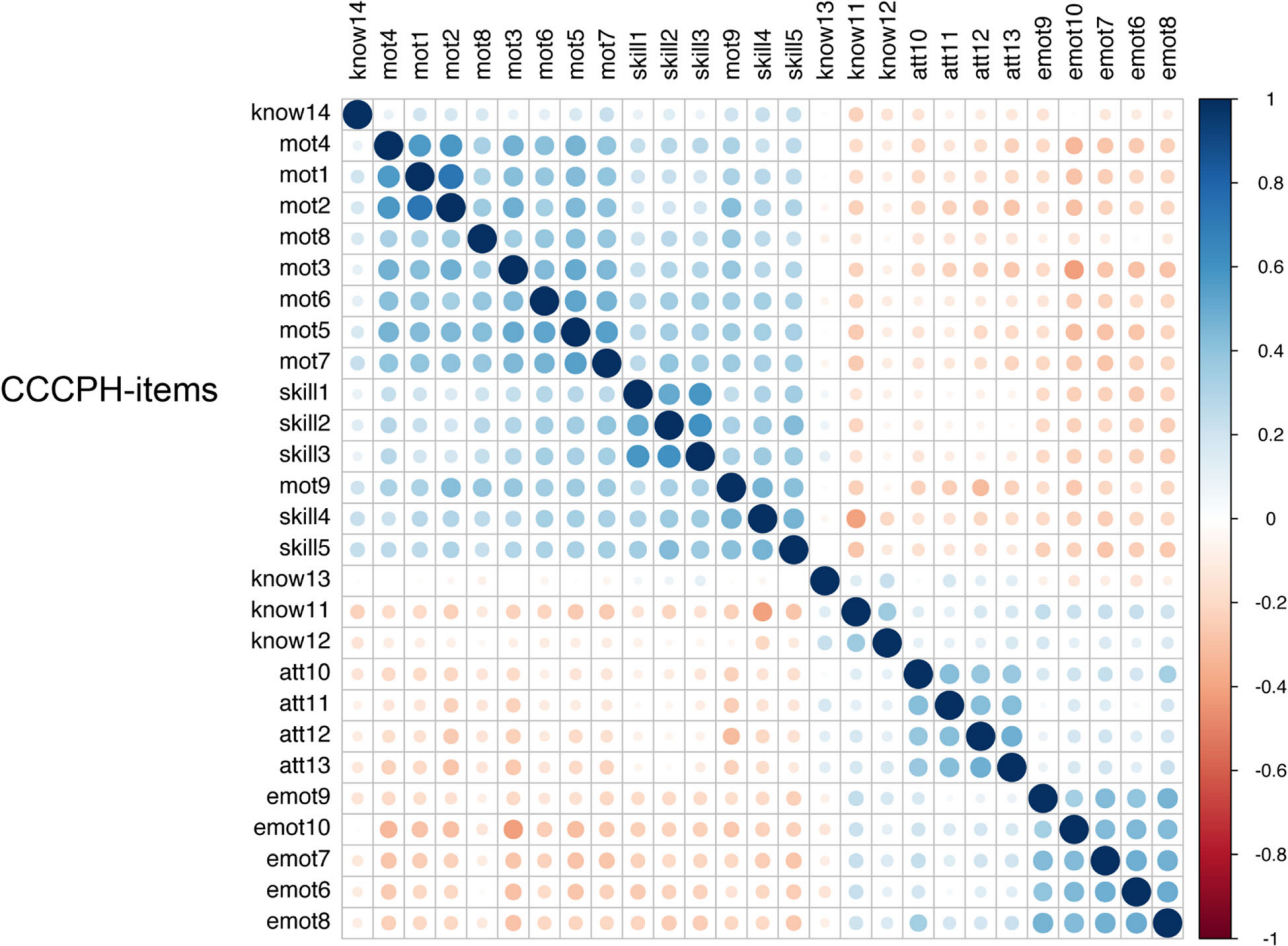
Correlations between CCCHP-items (blue circles are positive and red circles negative associations). The darkness of the colour represents the strength of the association (*N* = 759)

The participants (Table 2) were on average 35.4 years old in 2017 (p for difference < .001). There were more women than men (91% / 9%; p -value for difference was < 0.001). Most of the participants reported some specific multicultural training or education (mean 2.1). The participants perceived relatively seldom difficulties of patients (means 2.8 on 1 to 5 scale) and were relatively satisfied with their work (means 4.1 on 1 to 5 scale). There were no differences in any of the variables between men and women. The correlation matrices suggest that there were four of five patterns of associations between study variables suggesting four to five structural entities (Fig. 1). None of the original items were normally distributed and thus we evaluated using the R-package “bestNormalize” the best transformation to be used to normalize items. Based on this evaluation exp-transformation was used to all items. The original attitudes scale and knowledge scales were normally distributed (*p*-value for test 0.78 and 0.37) and the others not (all *p*-values < 0.001). Correlations between the dimensions were small or moderate (range from .07 to .54) and the strongest correlation was between motivation and skills –dimensions (Additional file 2).

**Table 2:** Sample characteristics (*N* = 759)

		Men		Women		
Variable	Range	Mean / N	(SD)/%	Mean / N	(SD) /%	*p*-value for difference
Age		35.1	(8.5)	35.0	(9.1)	0.387
Work tenure	1–6	3.6	(1.1)	3.4	(1.3)	0.233
Multicultural Education (1–3)	1–3	2.9	(0.6)	2.8	(0.6)	0.955
How often see patients from other cultures						
		6	(12.0)	44	(88.0)	0.718
		39	(8.9)	401	(91.1)	
		27	(10.0)	242	(90.0)	
Patient difficulty	1–5	3.00	(1.2)	2.8	(1.1)	0.268
Job satisfaction	1–5	4.2	(0.9)	4.1	(0.9)	0.566
Nativity	Finnish	65	(9.4)	624	(90.6)	0.878
	Foreign	7	(7.0)	63	(90.0)

**Fig. 2:**
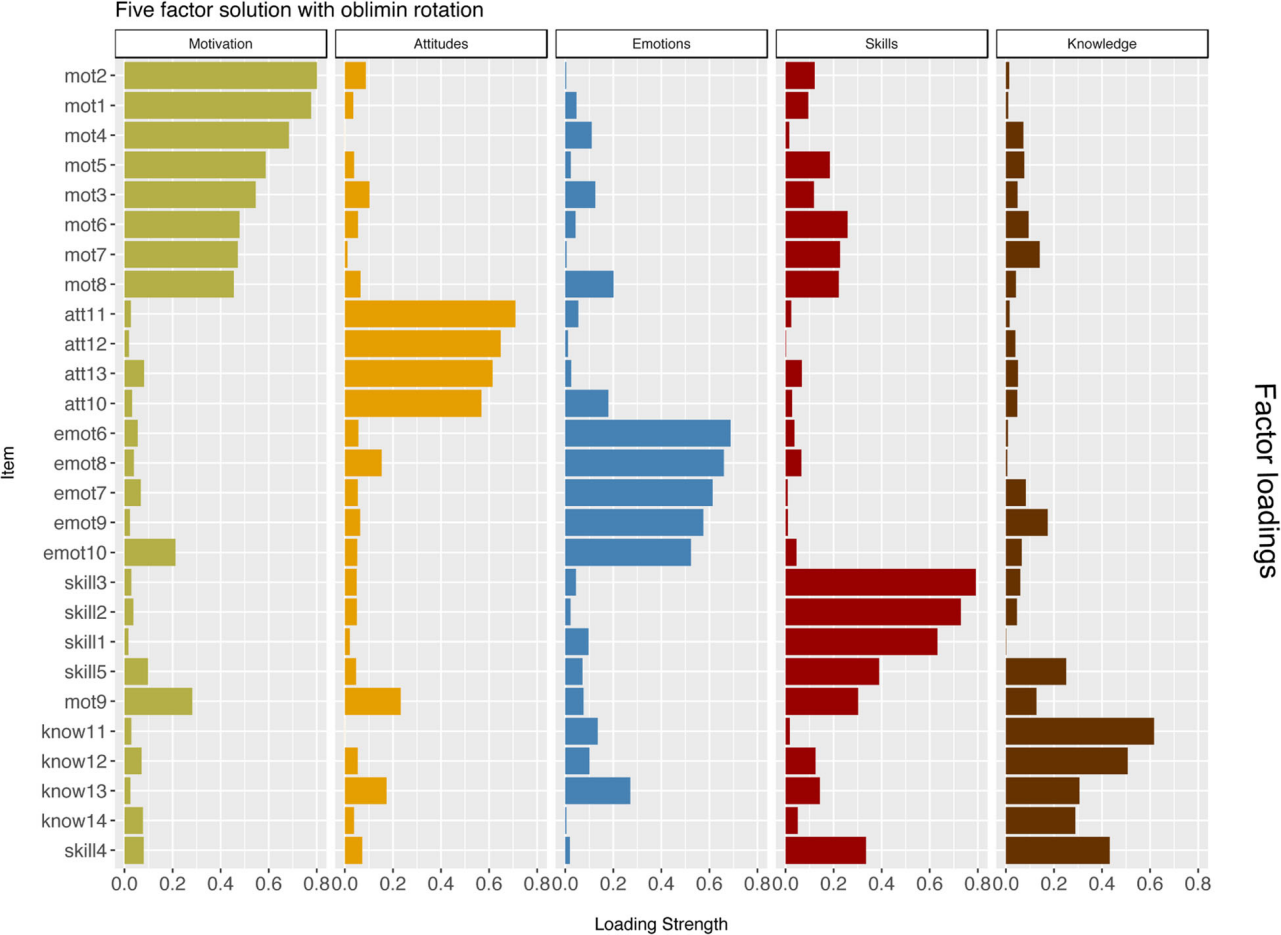
Factor loadings of CCCHP items in five-factor solution (Exploratory factor analyses with oblimin -rotation), *N* = 759

The exploratory factor analyses with oblimin rotation suggested that the original five-factor solution did not offer the best fit to the data following the eigenvalue > 1. The eigenvalues for the first factors were 8.6, 2.41, 2.20, 1.68, 1.44 and 1.03 suggesting six factor- solution. However, all the other methods supported the five-factor solution. In the five-factor solution most of the items had the strongest loading to their corresponding factors (Fig. 2). The notably exceptions were one of the motivation item (no 9) “It is important for me to treat patients according to their cultural needs and individual values” and one of the skill items (no 4, “Culturally specific factors of people [e.g. values, behaviour, norms, beliefs] influence their understanding of disease significantly, and should therefore be assessed and taken into consideration by healthcare professionals”). The loadings of items belonging to the knowledge factor were, however, in general slightly weaker than the loadings of other items to their corresponding factors. We repeated the factor analyses using the transformed items and the solution was very similar (Additional file 3). Furthermore, we repeated the EFA using the weighted least square estimator and the results did not change. The internal consistency (Cronbach’s alphas) was acceptable in all of the other sub-scales (range from .79 to .86) than in the knowledge sub-scale (.28).

**Fig. 3:**
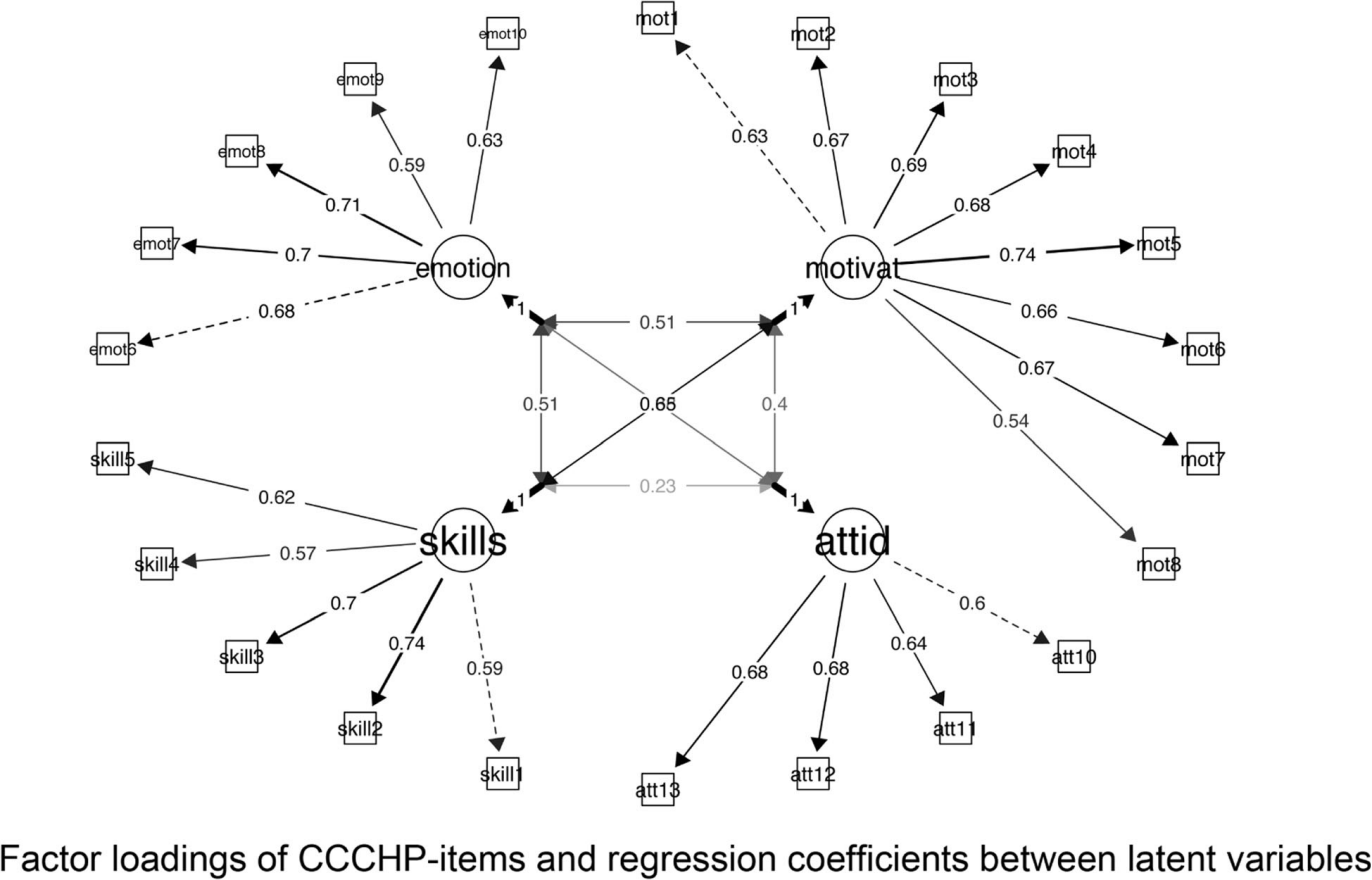
Final confirmatory four -factor solution of CCCHP-items (*N* = 759)

The confirmatory factor analyses with one underlying factor did not provide an acceptable fit to the data (χ^2^ (324) = 3145, CFI = .61 TLI = .58, RMSEA =0.104, AIC = 54,952). However, the solution with all items and five dimensions also did not provide an acceptable fit to the data (χ^2^ (309) = 1125.2, CFI = .89 TLI = .87, RMSEA =0.057, AIC = 52,862) and the items of the knowledge dimension loaded rather weakly to their corresponding latent variable. Modifications indexes suggested multiple cross-loadings and correlated errors between knowledge variables and variables of other constructs. Based on these suggestions, we dropped the knowledge dimension and one item from the motivation dimension (following the suggestions provided by the modification indexes) from the model and rerun the analyses. The four-factor solution provided a significantly better fit (χ^2^(198) = 616 CFI = 0.93, TLI = 0.92, RMSEA = 0.051, AIC = 42,492) to the data (Fig. 3). The Cronbachs’ alpha of the motivation scale without the one item was still .86, the skewness was slightly smaller (−.69 / -.66) and the correlation with other scales was the same (r -range from .52 to .13).

There were significant associations between all motivation/curiosity, attitude and emotions/empathy dimensions of cross-cultural competence and multicultural education received (Table 3) and the associations of education with motivation/curiosity, attitude and skills were robust to adjustment for age, gender, contacts with patients and work tenure. There were associations between all dimensions of cross-cultural competence and perceived difficulty of patients (Table 4) and these associations were all robust to adjustments for age, sex, contacts with patients and work tenure. Controlling the models for these factors attenuated the associations only marginally. The motivation/curiosity, skills and emotions/empathy dimensions of cross-cultural competence were associated with job satisfaction (Table 5) and all these associations remained significant after adjusting for age, sex, contacts with patients and work tenure.

**Table 3:** Associations between received education on cultural issues at work and cultural competence sub-scales, Linear regression coefficients (SE), (*N* = 759)

	Motivation	Attitude	Skills	Emotion
Education	0.08 ^***^	0.11 ^***^	0.04	0.06 ^*^
	(0.02)	(0.03)	(0.02)	(0.03)
N	759	759	759	759
R2	0.02	0.02	0.00	0.01
Step 2				
Age	0.05 ^*^	−0.06 ^*^	0.08 ^***^	0.05
	(0.02)	(0.03)	(0.02)	(0.03)
Gender	0.40 ^***^	0.12	0.24 ^**^	0.06
	(0.08)	(0.09)	(0.08)	(0.09)
Tenure	−0.01	0.01	−0.04	0.01
	(0.02)	(0.03)	(0.02)	(0.03)
Patient contacts	−0.04	0.06 ^*^	−0.03	− 0.07 ^*^
	(0.02)	(0.03)	(0.02)	(0.03)
Education	0.08 ^***^	0.10 ^***^	0.04	0.07 ^*^
	(0.02)	(0.03)	(0.02)	(0.03)
N	759	759	759	759
R2	0.06	0.03	0.04	0.02

*p < 0.05; **p < 0.01; ***p < 0.001

**Table 4:** Associations between perceived difficulty of patients and cultural competence sub-scales, Linear regression coefficients (SE), (*N* = 759)

	Model 1	Model 2	Model 3	Model4
Motivation	−0.17 ^***^			
	(0.04)			
Attitude		−0.16 ^***^		
		(0.04)		
Skills			−0.13 ^**^	
			(0.04)	
Emotion				−0.20 ^***^
				(0.04)
N	759	759	759	759
R2	0.02	0.02	0.01	0.03
Step 2				
Age	−0.10 ^*^	−0.12 ^**^	−0.09 ^*^	−0.10 ^*^
	(0.04)	(0.04)	(0.04)	(0.04)
Gender	−0.09	− 0.16	−0.14	− 0.17
	(0.14)	(0.14)	(0.14)	(0.14)
Tenure	−0.07	−0.07	− 0.08	−0.07
	(0.04)	(0.04)	(0.04)	(0.04)
Contacts with patients	−0.10 ^*^	−0.08	− 0.10 ^*^	−0.11 ^**^
	(0.04)	(0.04)	(0.04)	(0.04)
Motivation	−0.16 ^***^			
	(0.04)			
Attitude		−0.16 ^***^		
		(0.04)		
Skills			−0.12 ^**^	
			(0.04)	
Emotion				−0.20 ^***^
				(0.04)
N	759	759	759	759
R2	0.04	0.04	0.03	0.05

*p < 0.05; **p < 0.01; ***p < 0.001

**Table 5:** Associations between job satisfaction and cultural competence sub-scales. Linear regression coefficients (SE), (*N* = 759)

	Model 1	Model 2	Model 3	Model4
Motivation	0.12 ^***^			
	(0.03)			
Attitude		0.06		
		(0.03)		
Skills			0.15 ^***^	
			(0.03)	
Emotion				0.17 ^***^
				(0.03)
N	759	759	759	759
R2	0.01	0.00	0.03	0.03
Step 2				
Age	0.03	0.04	0.02	0.02
	(0.04)	(0.04)	(0.04)	(0.04)
Gender	−0.17	−0.11	−0.16	−0.11
	(0.12)	(0.12)	(0.12)	(0.12)
Tenure	0.01	0.01	0.02	0.01
	(0.04)	(0.04)	(0.04)	(0.04)
Contacts with patients	0.06	0.05	0.06	0.07 ^*^
	(0.03)	(0.04)	(0.03)	(0.03)
Motivation	0.13 ^***^			
	(0.04)			
Attitude		0.06		
		(0.04)		
Skills			0.16 ^***^	
			(0.04)	
Emotion				0.17 ^***^
				(0.03)
N	759	759	759	759
R2	0.02	0.01	0.03	0.04

*p < 0.05; **p < 0.01; ***p < 0.001

## Discussion

This study examined psychometric properties of a Finnish version of the original questionnaire (CCCHP) [11] by measuring cross-cultural competence in a representative sample of Finnish registered nurses. Our results suggested that the solution with four dimensions (motivation/curiosity, attitudes, skills and emotions/empathy) offered the best fit to the data. Our data showed satisfactory internal consistency of the four scales, and confirmatory factor analyses resulted in a good model fit to the data with the four-factor solution. The main problem was that the original items were not normally distributed and the items theoretically belonging to the knowledge dimension did not seem to form a coherent latent construct.

Moreover, all scales were found to be associated with the education received and all but attitude with perceived difficulty of patients and better job satisfaction supporting appropriate criterion validity of the measurement. The low explained variance (R^2^) in the regression models suggest that competence scales are not able to explain large share of the variance in the outcome variables, but that is of course quite understandable. The outcomes used as concurrent validity measures are multifactorial and relatively weak associations were to be expected. More studies supporting the four-factor structure are needed to confirm whether the shorter instrument, suggested in this study, is replicable and provides efficient predictive validity and invariance over time.

Regarding the dimensions of cross-cultural competence, our results highlight the importance of the affective component (i.e. the emotion/empathy dimension). This is understandable as emotions are in many ways at the center of our everyday lives. Emotions are closely and strongly linked with our perceptions and may have a strong link with motivation systems [27]. Encountering patients from different cultures awakes affective states, which reveal how well or poorly a professional is doing in understanding and advancing the patients’ situation. Feeling frustrated or having difficulties in situations with foreign patients may ultimately affect quality of care and also the professionals’ own well-being. Our recent study [22] has shown that of all four dimensions of cross-cultural competence, only the emotions/empathy dimension was associated with perceived time pressure and psychological distress among native and foreign-born nurses. Further research is needed to explore the role of emotions in cross-cultural competence and to test whether the affective dimension is equally important in countries where diversity issues differ from Finland and Germany (see also [11]).

The cognitive component (i.e. the knowledge/awareness dimension) did not fit well to the data defined by the model in the Finnish version of the CCCHP and thus did not form a coherent dimension of its own in the model. This might not implicate, however, that having cross-cultural knowledge is not of importance in the Finnish context. The question might be more on how the components are defined and measured. Awareness and knowledge are both regarded as the most important elements of cross-cultural competence (together with cultural skills) but in some models the two are combined together [4]. In the CCCHP, only one of the four items of knowledge/awareness dimension was originally associated with professionals’ self-awareness, while the other items represented knowledge. Also, the knowledge/awareness subscale showed the lowest reliability (measured by Cronbach’s alpha) in the Bernhard’s study. It is possible that the combination of the items or the content of single items were not comprehensive enough to tap on the main idea of cultural knowledge and awareness and thus this dimension did not emerge in this study.

### Limitations

Several limitations need to be kept in mind when interpreting the results of this study. First, the instrument of cultural competence was self-reported and thus based on individuals’ perceptions, which are susceptible to social desirability effects. As immigration policies in Finland and elsewhere are also political issues, the participants may have hesitated to answer questions on these issues and thus the answers they gave might not have reflected their true perceptions [8, 28]. We tried to minimize this bias by taking care that the respondents knew that their anonymity was maintained throughout the whole process of collecting, analysing and reporting data. Another problem associated with evaluating the respondent’s answers is a finding that people tend to overestimate their cultural competence [29], which might then mislead the managers to believe that their workers’ cultural competence is at a good level and therefore needs no improvement. Hence, in order to obtain more objective data on cultural competence in the future, different measurements using quantitative and qualitative methods are needed [8]. For example, direct observation in clinical interactions between healthcare professionals and patients from different cultures could be used. Another option is to collect information on the usage of available tools made for healthcare professionals (an example of this kind of tool is the Cultural Formulation Interview added to the Diagnostic and Statistical Manual of Mental Disorders (DSM-5)) or comparing healthcare professionals’ own perception with perceptions of his or her immediate colleagues as well as patients’ perceptions.

Second, a relatively low response rate in both of the samples limits the generalizability of our findings. However, the guideline of Nulty [30] indicates that the response rate in our study was sufficient for the number of participants in the initial sample.

### Implications for practice

This revised version of the cross-cultural competence in healthcare instrument can be a useful tool for collecting information on an individual level as well as part of larger interventions aiming to improve cross-cultural competence at a hospital level. The benefits of using this instrument in practice are that the same questionnaire is eligible for the largest occupational group (nurses) and fulfilling the questionnaire (with its 22 items) is much less time-consuming to busy healthcare workers compared to many other cross-cultural competence measurements (with up to 80 items or more). Healthcare professionals can use the questionnaire to reflect their own attitudes, emotions, skills, and motivation in working with patients from different cultures. Healthcare organizations can collect data from their professionals and use it as a part of improving quality of patient care and encouraging their workers to develop their professional competence regarding interactions with patients from different cultures (see also [11].

The instrument (both the Finnish version as well as the original German version) seems to be most suitable for healthcare units that do not focus only on foreign-origin patients because some of the questions in the instrument use comparisons of perceptions between treating patients from one’s own cultural background and foreign background. Thus, professionals working solely with, for example, immigrants might find questions such as “I prefer treating patients from my own cultural background than those who seem foreign to me” strange and not applicable to their work.

## Conclusions

The revised Finnish version of the CCCHP provides a useful tool not only for studying various aspects of cross-cultural competence in nurses, but also for healthcare organizations trying to monitor and improve cross-cultural competence of their personnel. The latter is specifically important because the personnel’s cross-cultural competence is one of the key requirements in delivering effective and culturally sensitive healthcare services for patients from different cultures. In line with Bernhard et al. [11], we suggest that future research should explore more the role of professionals’ affective states in the context of cross-cultural encounters. For example, uncovering possible negative emotions related to cross-cultural encounters might give insight on how to arrange healthcare working conditions to better support effortless interactions between healthcare professionals and foreign patients.

## Additional files

Additional file 1: **Figure S1.** Missing pattern. (DOCX 106 kb)

Additional file 2: **Figure S2.** Correlations between original CCCHP- scales. (DOCX 90 kb)

Additional file 3: **Figure S3.** Exploratory factor analyses using exp-transformed items. (DOCX 110 kb)

## Abbreviations

CCCHP: The Cross-Cultural Competence instrument of Healthcare Professionals
COPE: Competent workforce for the future –project
DSM-5: Diagnostic and Statistical Manual of Mental Disorders
