# Supplementary figures and images for: Testing the psychometric properties of the Finnish version of the cross-cultural competence instrument of healthcare professionals (CCCHP)
Source: BMC Health Serv Res. 2019 May 8;19:294. doi: 10.1186/s12913-019-4105-2 (PMC6505538; doi:10.1186/s12913-019-4105-2)

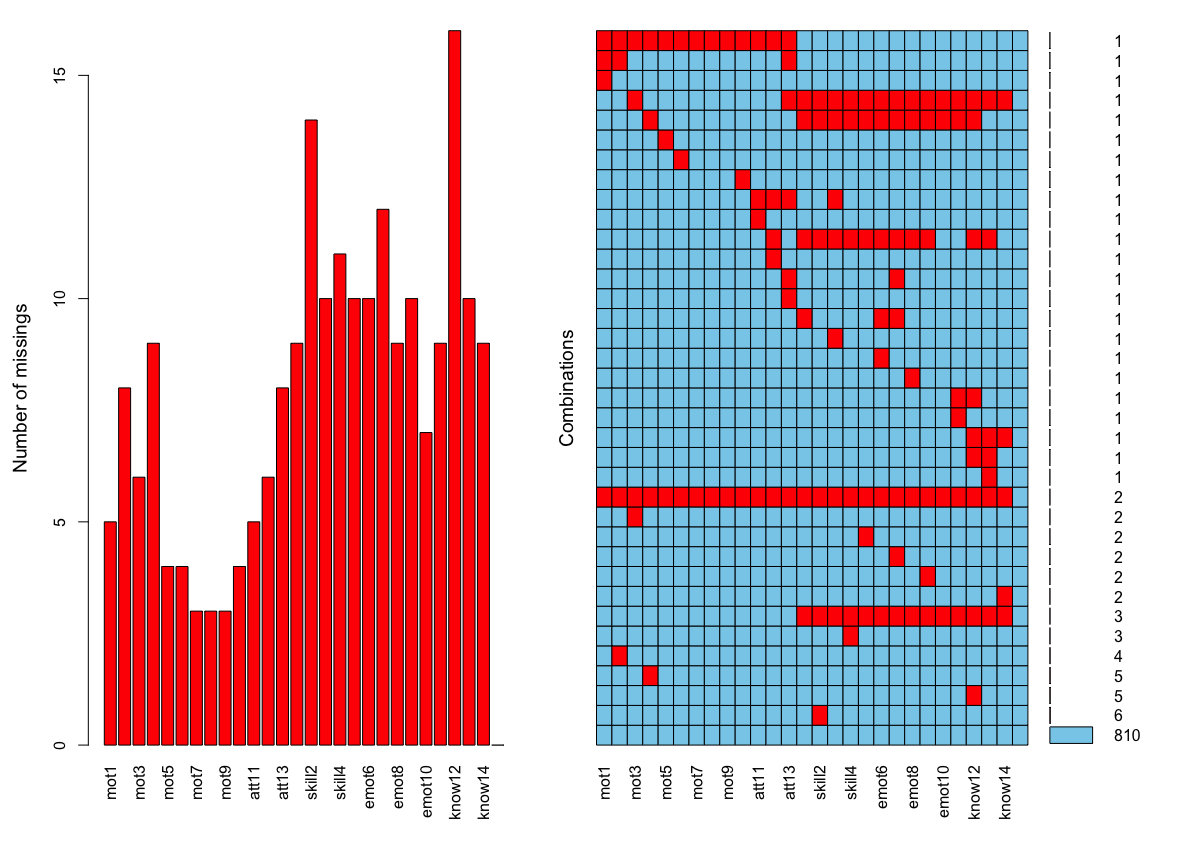


Figure S1. Missing pattern.

Supplement: Supplementary file 1 — Figure S1. Missing pattern. (DOCX 106 kb) [file 12913_2019_4105_MOESM1_ESM.docx]

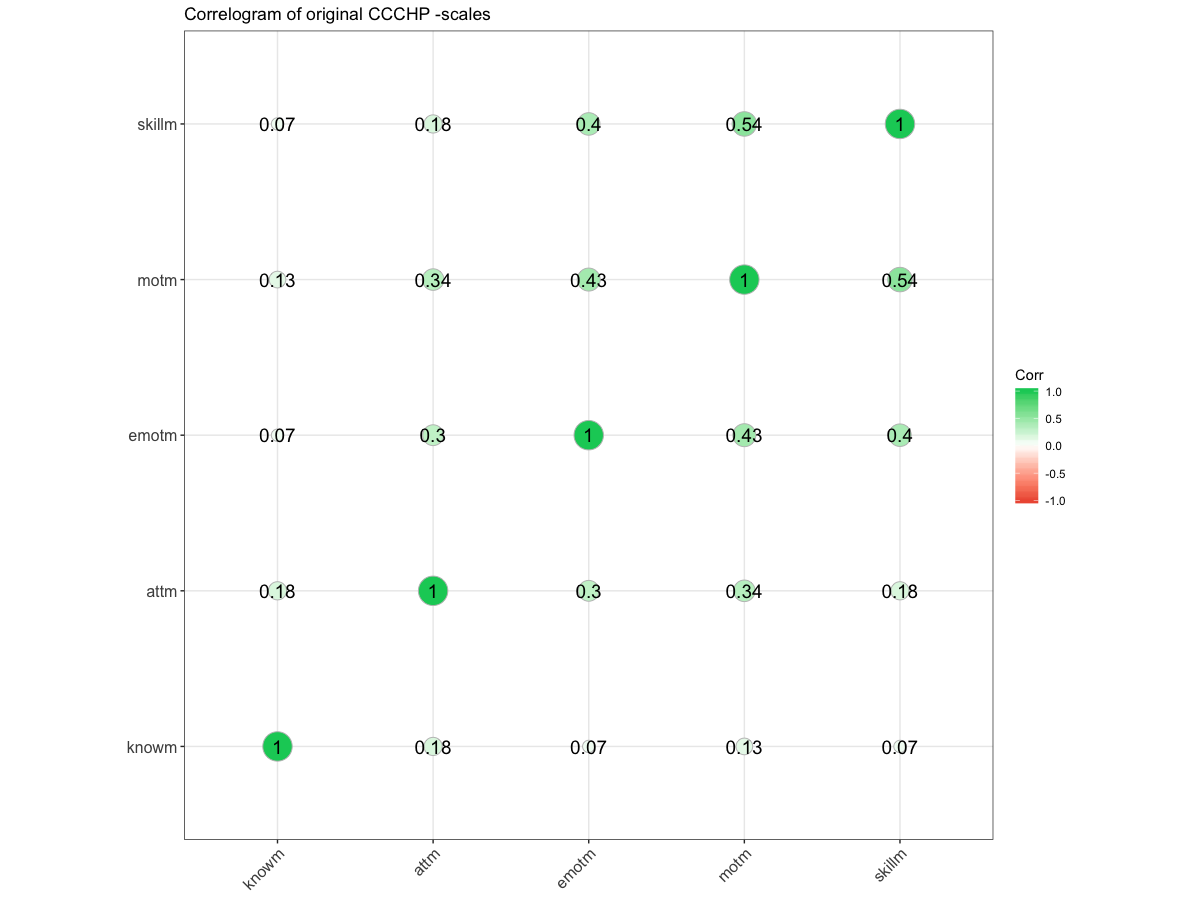


Figure S2. Correlations between original CCCHP- scales.

Supplement: Supplementary file 2 — Figure S2. Correlations between original CCCHP- scales. (DOCX 90 kb) [file 12913_2019_4105_MOESM2_ESM.docx]

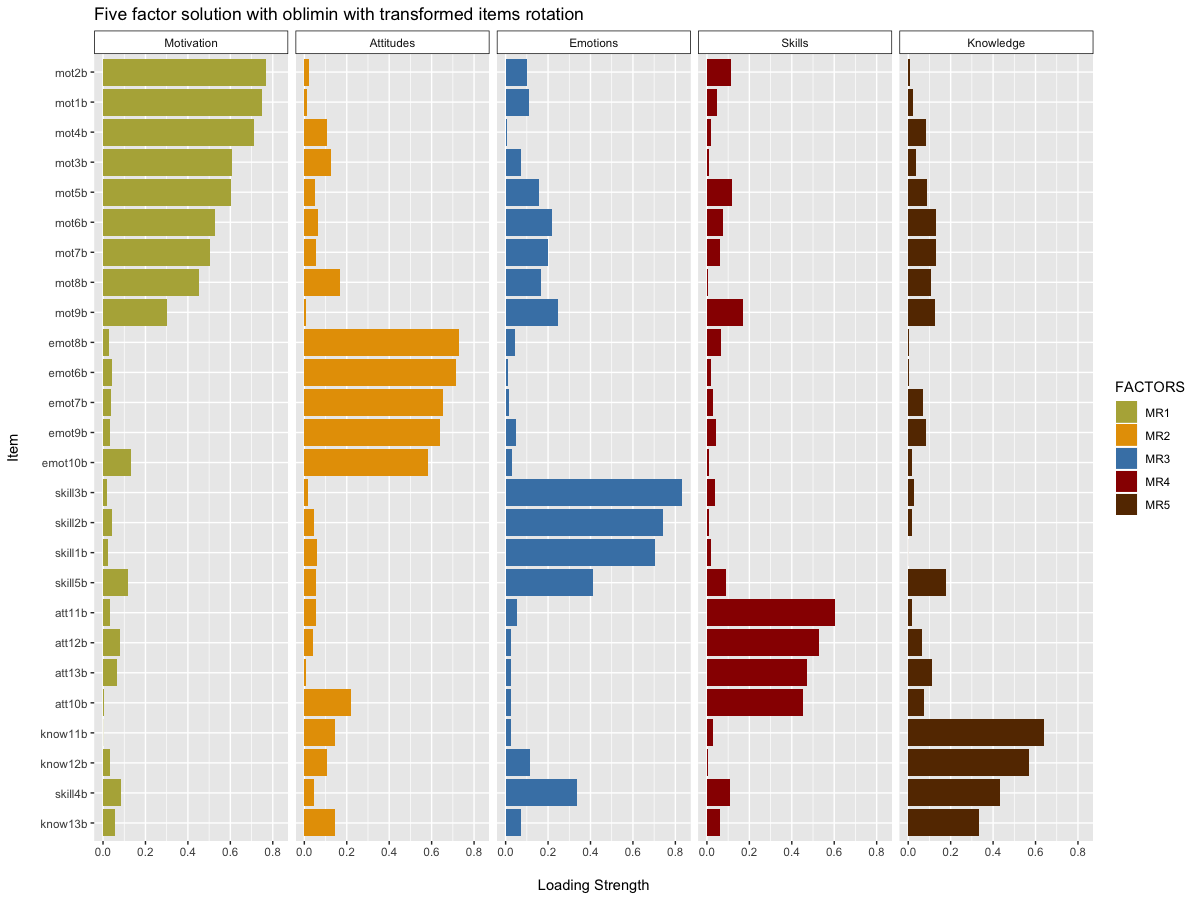


Figure S3. Exploratory factor analyses using exp-transformed items.

Supplement: Supplementary file 3 — Figure S3. Exploratory factor analyses using exp-transformed items. (DOCX 110 kb) [file 12913_2019_4105_MOESM3_ESM.docx]
